# Pattern of neurological disease seen among patients admitted in tertiary care hospital

**DOI:** 10.1186/1756-0500-7-202

**Published:** 2014-03-31

**Authors:** Rajib Nayan Chowdhury, ATM Hasibul Hasan, Yusuf Ur Rahman, Shafikul Islam Khan, Ahmed Riyad Hussain, Shamim Ahsan

**Affiliations:** 1 Dhaka Medical College Hospital, Dhaka, Bangladesh; 2 Department of Medicine, Dhaka Medical College Hospital, Dhaka, Bangladesh

**Keywords:** Neurologic disease

## Abstract

**Background:**

Neurologic disorders are not uncommon at in patient departments of different hospitals. We have conducted the study to see the pattern and burden of neurologic disorders at different inpatient departments of a tertiary care centre.

**Methodology:**

This retrospective observational study was carried out from the records and referral notes of neurology department of Dhaka Medical College Hospital (DMCH) from July 2011 to June 2012. A total 335 patients were evaluated by consultant neurologists during this period.

**Result:**

Majority of the patients (59.7%) presented after the age of forty years. The mean age at presentation was 45.11 ± 17.3 years with a male predominance (63.3%). Stroke was the most common condition (47.5%) observed at referral, followed by seizure (9.3%), disease of spinal cord (7.8%) and encephalopathy (6.3%). Even after consultation, 30 patients remained undiagnosed and 6 were diagnosed as functional disorder. Department of Medicine (231, 69%) and Cardiology (61, 18.2%) made most of the calls. More than half (56%) of the stroke patients were referred from medicine and one third (35.2%) from cardiology. Seizure (67.7%), problem in spinal cord (92.3%), coma (50%), encephalopathy (57.1%), motor neuron disease (MND) (72.7%) were common reasons for referral from department of Medicine. Whereas patients with cord disease (7.3%), CNS tumor (40%), seizure disorder (6.5%) and stroke (3.8%) were referred from surgery. Department of Obstetrics and Gynecology sought help for stroke (2.5%), seizure (12.9%), MND (27.3%), coma (16.7%) and encephalopathy (9.5%).

Hypertension, diabetes, ischemic heart disease, dyslipidaemia and respiratory problem were significantly associated co-morbid conditions in stroke patients (at 95% CI, *p* value is <0.001, <0.01, <0.001, <0.05, <0.05 respectively). Hematological disorders were common association among patients with cord problem (<0.05).

**Conclusion:**

Wide ranges of neurological problems are often managed by physicians and surgeons, especially those from medicine and cardiology. Where ever available consultation from neurologists can help in diagnosing and managing these cases.

## Background

The overall global burden of neurologic disease is approximately 20%, the majority being in the developing countries [1]. The incidence of neurologic disorder in UK is 0.6% with an overall 6% lifetime prevalence rate [2]. Many patients with acute or chronic neurological problems often get admitted under general medicine and other departments. Some with other primary diagnosis may also develop neurological complication due to course of illness or as a part of complication. Approximately 15-20% of all medical admission and 40% inpatients in medical wards are neurologic problems that often require consultation even in UK [3-6]. Despite these statistical logics whenever available, expert neurologic assessment and management can alter the working diagnosis and can have a positive impact on overall hospital management [7]. Diagnostic errors by non neurologists are not very uncommon, especially regarding epilepsy and other non-organic illness [8,9]. Retrospective studies have also shown that diagnostic change following neurology consultation is seen in 32-50% of inpatient referrals [10-12]. Studies have also proved that a liaison with neurology consultation may improve inpatient care in UK [9,13].

Neurology was introduced as a specialty in Bangladesh during the 1960s’. Apart from infection and malnutrition, an excess burden of cerebrovascular disease and stroke at an early age denotes a higher risk of mortality and morbidity in Bangladesh [14,15]. As neurologic disorders are quite common among all medical admissions in DMCH and there are lack of facilities elsewhere in the country, varieties of neurological problems are referred to Dhaka Medical College Hospital (DMCH). Previously we did not have any published data regarding the neurology consultation pattern in Bangladesh. The available data on this issue are mostly from European countries. We therefore tried to audit the nature of neurologic involvement among patients admitted in different departments and liaison of neurology with these departments through this referral system.

## Methods

This is a retrospective observational study. We reviewed the records and referral notes from Neurology department of Dhaka Medical College Hospital (DMCH) from July 2011 to June 2012. Dhaka Medical College Hospital, the centre of excellence and one of the highest centers of referral for any health related issue has 1600 inpatient beds. In addition to Department of Medicine, Surgery, Obstetrics and Gynaecology, the hospital has enriched departments like Neurology, Cardiology, Gastroenterology etc. Neurology department has 22 inpatient beds. Our study population included 335 patients from the hospital records over the period of one year. All new inpatient referrals to neurology in DMCH were eligible for inclusion. Each of the referred patients was examined by the Consultant Neurologist who attended the referral. Patient’s problem was diagnosed clinically with proper history, examination and with help of investigations in required cases. Information regarding the demographic and clinical profile was gathered through a questionnaire. Neurological diagnosis was categorized under 12 broad headings (Table 1). The research protocol was reviewed and accepted by the ethical review committee of Dhaka Medical College. Analysis was done using SPSS version 16.0. At 95% CI, *p* value <0.05 was considered significant.

**Table 1:** Distribution of diseases from different departments

**Disease**	**Number & percentage of patients from different departments**					
**Diagnostic break down (number, %)**	**Medicine**	**Surgery**	**Obs & Gynae**	**Cardiology**	**ICU**	**Burn and plastic surgery**
Stroke (159, 47.5)	89(56)	6 (3.8)	4(42.5)	56(35.2)	2(1.3)	2(1.3)
Seizure (31,9.3)	21(67.7)	2(6.5)	4(12.9)	0	0	4(12.9)
CNS infection (13, 3.9)	13(100)	0	0	0	0	0
Peripheral neuropathy (13, 3.9)	13(100)	0	0	0	0	0
GBS (3, 0.9)	3(100)	0	0	0	0	0
MND (11, 3.3)	8(72.7)	0	3(27.3)	0	0	0
Cord disease (26, 7.8)	24(92.3)	2(7.3)	0	0	0	0
Dementia (5, 1.5)	5(100)	0	0	0	0	0
Tumor (5, 1.5)	3(60)	2(40)	0	0	0	0
Coma (12, 3.6)	6(50)	0	2(16.7)	0	4(33.3)	0
Encephalopathy (21, 6.3)	12(57.1)	4(19)	2(9.5)	3(14.3)	0	0
Functional (6, 1.8)	6(100)	0	0	0	0	0
Undiagnosed clinically (30, 9)	28(93.3)	0	2(6.7)	0	0	0
Total number and (%) of patients within different departments	231 (69%)	16 (3.2%)	15 (3%)	61 (18.2%)	6 (1.8%)	6 (1.8%)

## Results

A total of 335 patients were seen by neurologists within this period of time. Most of the patients (59.7%) presented after the age of forty years. Only 2 (0.6%) patients were below 10 years. The distribution of patients were more or less similar in different age groups from 21 years to those above 60 years (15-18%). The mean age at presentation was 45.11 ± 17.3 years. The male patients (63.3%) predominated with a sex ratio was almost 2:1 (Table 2). Stroke was the most common condition (149, 47.5%) observed at referral, followed by seizure (31, 9.3%), disease of spinal cord (26, 7.8%) and encephalopathy (21, 6.3%). But CNS infection (3.9%), peripheral neuropathy (3.9%), coma (3.6%) and MND (3.3%) were less common. Conditions like dementia (1.5%), tumor (1.5%) and GBS (0.9%) were rarely consulted. Even after consultation, 30 patients remained undiagnosed at first visit and 6 patients had non organic (functional) diagnosis (Table 1). Department of Medicine was the largest consultation seeker (231, 69%), followed by department of Cardiology (61, 18.2%). Number of patients referred from Surgery (16), Obstetrics and Gynecology (15), ICU (6) and Burn & Plastic Surgery (6) were very few. More than half (56%) of the stroke patients were referred from medicine and one third (35.2%) from cardiology. Seizure (67.7%), problem in spinal cord (92.3%), coma (50%), encephalopathy (57.1%) MND (72.7%) were common reason for referral from department of Medicine. Some diseases like CNS infection, peripheral neuropathy, GBS, dementia were referred only from this department. Department of Cardiology took help only for stroke (35.2%) and encephalopathy (14.3%). Patients with cord disease (7.3%), CNS tumor (40%), seizure disorder (6.5%) and stroke (3.8%) were referred from surgery. Department of Obstetrics and Gynecology sought help for stroke (2.5%), seizure (12.9%), MND (27.3%), coma (16.7%) and encephalopathy (9.5%) (Table 1).

**Table 2:** Socio demographic profile of the patients (N = 335)

**Parameter**		**n**	**%**
	**Age**		
	0-10 yr	2	0.6
	11-20 yrs	25	7.5
	21-30 yrs	56	16.7
	31-40 yrs	52	15.5
	41-50 yrs	87	26
	51-60 yrs	62	18.5
	>60 yrs	51	15.2
	**Sex**		
	Male	212	63.3
	Female	123	36.7

Table-2: Shows the age group and sex distribution of patients. Most common age of presentation was after 40 yrs.

Hypertension, diabetes, ischemic heart disease, dyslipidaemia and respiratory problem were significantly associated co-morbid conditions in stroke patients (at 95% CI, *p* value is <0.001, <0.01, <0.001, <0.05, <0.05 respectively. Hematological disorders (especially disorders with extramedullary haematopoiesis) were common association among patients with cord problem (Table 3).

**Table 3:** Common co-morbidities observed among patients

	**Percentage of patient with co-morbidity**							
**Disease**	**HTN**	**DM**	**IHD**	**Arryhtmia**	**CKD**	**Dyslipidaemia**	**Respiratory disorder**	**Haematological disorder**
Stroke (159)	83.3	100	85.2	66.7	66.7	100	33.3	0
Seizure (31)	2.1	0	0	0	0	0	0	20
CNS infection (13)	0	0	0	0	9.5	0	16.7	0
Peripheral neuropathy (13)	0	0	0	0	0	0	16.7	0
GBS (3)	0	0	0	0	0	0	0	0
MND (11)	0	0	0	0	0	0	0	0
Cord disease (26)	0	0	2.5	0	0	0	16.7	50
Dementia (5)	3.1	0	6.2	0	0	0	0	0
Tumor (5)		0	0	0	0	0	16.7	0
Coma (12)	2.1	0	2.5	33.3	9.5	0	0	0
Encephalopathy (21)	2.1	0	3.7	0	0	0	0	0
Functional (6)		0	0	0	0	0	0	0
Undiagnosed clinically (30)	7.3	0	0	0	14.3	0	0	30
Total number of patients with co-morbidities	96	16	81	6	21	16	12	10
*P* value	<0.001	<0.01	<0.001	>1	> 0.05	<0.05	<0.05	<0.001

## Discussion

Though the number of neurologists (only 86) [16] has increased over last decade in Bangladesh, it is still not enough. Many patients with neurological problems are often dealt by internists and others from different specialties. Studies have proved the usefulness of liaison with neurology, especially in teaching hospitals [4,11,13] and the patient care also improved with specialist management. The length of hospital stays was also shortened in study by Forbes et al [4]. Moeller et al [17] also provided enough data to prove the reliability of initial neurology referral. But we didn’t have any similar study in our setting. So we tried to get a glimpse of our neurology service in this teaching hospital. This paper represents a comprehensive survey of inpatient admission of neurological problems and referral seeking behavior of different departments. It gives a transparent idea about the burden of neurological cases in these departments.

The patient demographic profile reflects that most of the referral and consultation sought was from the older (the mean age, 45.11 ± 17.3 years) and male patients. The distribution was similar to Gajurel et al [18] from India. Probably the age and sex related increase in cerebrovascular event has made the neurological problems a common ailment in many studies including this one [7,12]. A total of 33226 patients were admitted in Department of Medicine of DMCH, the largest fraction (14.3%) of which was stroke [19] and not surprisingly the most common cause of referral, irrespective of the departments was stroke (47.5%) which is also similar (49.5%) to the report of Gujral et al [18]. But the total burden of neurological problem in Medicine is not known. Though most (231) of the patients were referred from the department of medicine to neurology, it accounts for only 0.69% of all inpatient admissions [19]. Even the data from death and discharge profile of The Royal College of Physicians of London, revealed that the majority of patients with serious neurological disease were under the care of physicians (and sometimes surgeons) in disciplines other than neurology [20]. A significant portion of referral (18.2%) came from department of cardiology, especially for patients with stroke (35.2%), which can be explained by the common patho-physiology of vascular events. Stroke as being major killer and cause of disability in developing world like us, it was the commonest cause for referral. Stroke causes 1.6 million death in china and 0.6 million death in India [21]. Other than stroke, seizure (9.3%) was the next common event for consultation seeking. In contrary to this most the European studies except for the Irish, reported epilepsy as a major cause of referral, followed by stroke [7,19,22,23]. The Irish reporting had stroke (22.7%) and epilepsy (10.2%) as two most common causes for referral. This is probably partly due to the difference in disease prevalence and the methodology applied in these studies. Although, the annual incidence of epilepsy (50 per 100000 population) in developing countries is twice that of the developed world [24], the increased burden of stroke has outnumbered other diseases in our study. The frequency of other neurological problems like, cord disease, encephalopathy, CNS infection, peripheral neuropathy was very low. The pattern these less common diseases, was similar to the above mentioned European studies [7,19,22,23].

Seizure was also common cause of referral from the department of Obstetrics & Gynecology and Burn & Plastic surgery. This is mostly due to referral from eclampsia ward and that the patients with epilepsy often encounter burn accidentally during a seizure event. Unfortunately even after the efforts by neurologists about 9% of the patients remained undiagnosed. Hypertension, DM, ischemic heart disease, dyslipidaemia, hematological disorder and respiratory disorders were significant (p value <0.05) association with neurological problems. All these are common comorbidities for vascular events eg stroke [25].

We had some limitations in this study. First of all, patients were seen only once by the neurologist. So follow up data was unavailable. Secondly, each patient was consulted by a single neurologist. So there is chance of diagnostic variability. Further studies involving large cohorts and cross checking neurologists are required to validate these findings.

## Conclusion

A significant portion of neurological workload is still managed by non neurologist physicians and the pattern of disease includes the entire possible range of neurological problems. The neurologists can make a valuable contribution to the diagnosis and management of neurological problems through the medium of neurological ward referrals.

## Competing interests

The authors declare that they have no competing interests.

## Authors’ contributions

RNC was involved concept, data collection and revision of manuscript for this study. ATMHH was involved in data analysis and writing the article. The rest were involved in design, data collection and analysis. All the authors have read and approved the final version of the manuscript.
